# Socioeconomic Deprivation Is Not Associated with Outcomes after Esophagectomy at a German High-Volume Center

**DOI:** 10.3390/cancers15102827

**Published:** 2023-05-18

**Authors:** Marius Kemper, Jana Zagorski, Jonas Wagner, Julia-Kristin Graß, Jakob R. Izbicki, Nathaniel Melling, Stefan Wolter, Matthias Reeh

**Affiliations:** Department of General, Visceral and Thoracic Surgery, University Medical Centre Hamburg-Eppendorf, 20251 Hamburg, Germany

**Keywords:** esophageal cancer, esophagectomy, socioeconomic deprivation, outcome

## Abstract

**Simple Summary:**

It is known that socioeconomically disadvantaged people more often develop esophageal cancer. Therefore, we assumed that those patients more often have advanced tumor stages and comorbidities at the time of surgery and, thus, are more likely to suffer from postoperative complications and poorer survival. To clarify this, we used the purchasing power of the respective postal codes to estimate the socioeconomic status (SES) of 310 patients who had undergone surgery for esophageal cancer in our institution. Fortunately, it turns out that SES was not associated with tumor stage or comorbidities at the time of surgery. Moreover, SES was neither related to postoperative complications nor overall survival. In conclusion, socioeconomic inequalities of patients treated at a high-volume center do not affect treatment results.

**Abstract:**

In Germany, socioeconomically deprived citizens more often develop esophageal carcinoma, since typical risk factors follow the social gradient. Therefore, we hypothesized that socioeconomic deprivation might also be associated with advanced tumor stages and comorbidities at the time of surgery. As a consequence, socioeconomic deprivation may be related to postoperative complications and reduced overall survival. Therefore, 310 patients who had undergone esophagectomy for cancer in curative intent between 2012 and 2020 at the University Medical Center Hamburg-Eppendorf (UKE) were included in this study. Socioeconomic status (SES) was estimated using the purchasing power of patients’ postal codes as a surrogate parameter. No association was found between SES and tumor stage or comorbidities at the time of surgery. Moreover, SES was neither associated with postoperative complications nor overall survival. In conclusion, socioeconomic inequalities of patients treated at a high-volume center do not affect treatment outcomes.

## 1. Introduction

Esophageal carcinoma is among the most common causes of cancer deaths in Germany. Each year, approximately 5710 new diagnoses are made in men and about 1840 in women. Unfortunately, most patients present at advanced stages and die in the course of the disease [1]. It has been demonstrated that not only in Germany [2] but also in other countries, e.g., Sweden [3], France [4] and Iran [5], esophageal carcinomas occur more frequently in socioeconomically deprived people.

The two main histological subtypes, squamous cell carcinoma and adenocarcinoma, have different risk factors. Among the most critical risk factors for esophageal squamous-cell carcinoma are nicotine and alcohol consumption. Obesity and the frequently associated gastroesophageal reflux disease favor the development of esophageal adenocarcinoma. In Germany, these risk factors are related to socioeconomic status (SES): people with a high SES more often exceed the recommended moderate amounts of alcohol [6] and economically weaker populations smoke more often and are less successful at quitting [7]. The prevalence of obesity also follows the social gradient: it is more common in people with low SES [8].

The treatment of esophageal cancer is complex and depends on the histologic subtype, tumor stage, and the patient’s operability. Endoscopic resection is performed in patients with an early carcinoma (Union for International Cancer Control (UICC) TNM stage cT1a, N0, M0) [9]. Operable patients with stage cT1b-T2 N0 primarily undergo a transthoracic radical en bloc esophageal resection with systematic lymph node dissection [9]. Neoadjuvant radiochemotherapy is recommended in patients with locally advanced esophageal squamous cell carcinoma (cT3-T4 or N1-N3 M0). In patients with locally advanced esophageal adenocarcinoma (cT3-T4 or N1-N3 M0), perioperative chemotherapy is recommended [10]. Regarding the surgical technique, open esophagectomy was the standard for decades. However, in recent years, laparoscopic, hybrid, and cost-intensive robotic esophagectomies have become well-established due to better short-term outcomes [11].

In Germany, health insurance is mandatory and provides comprehensive medical care, including state-of-the-art cancer treatment, to all citizens and legal residents. It is financed by statutory health insurance (SHI) and private health insurance (PHI). Around 85% of the population is covered by SHI, which is mandatory for individuals with an income below a certain threshold. Those who earn above this threshold can choose between SHI and PHI. Contributions of PHI members depend on their age, health status, and level of coverage [12]. Since the amount paid by PHI to the hospital is usually a multiple of the amount paid by SHI, privately insured patients often have advantages; for example, shorter waiting times for appointments.

Given this background, we hypothesized that, in Germany, esophageal cancer is not only characterized by a social determination of incidence, as shown by Hoebel et al. [2], but also with advanced tumor stages and comorbidities at the time of surgery. Thus, the risk for postoperative complications might be higher, and, as a consequence, the overall survival of socially deprived patients might be reduced. Unfortunately, due to the lack of digitalization and data exchange in the German health care system, there is no detailed German national register that captures the relevant confounders, such as comorbidity and surgical technique, to adequately clarify whether the SES is associated with treatment outcome after oncologic esophagectomy. Therefore, taking into account the relevant confounders, this retrospective single-center study is the first to investigate whether socioeconomic status is associated with treatment results after esophagectomy for cancer in Germany. If our hypothesis proves true, targeted prevention programs should be developed and provided.

## 2. Materials and Methods

### 2.1. Patient Selection

Inclusion criteria were (I) histologically confirmed esophageal malignancy with UICC stage I-III at the time of diagnosis and (II) transthoracic radical en bloc esophageal resection with systematic lymph node dissection between 2012 and 2020 at the Department of General, Visceral and Thoracic Surgery, University Medical Center Hamburg-Eppendorf (UKE). Patients with a history of former carcinoma were excluded. In total, 369 individuals have been assessed for eligibility. Of these, 35 patients have been noted as ineligible. Twenty-four individuals were eligible but refused to enter the study. A total of 310 patients were confirmed eligible and recruited.

### 2.2. Treatment

According to the German guideline for esophageal cancer [13], patients with cT1b-T2 N0 M0 carcinomas primarily underwent transthoracic radical en bloc esophageal resection with systematic lymph node dissection. Patients with locally advanced, non-metastatic esophageal squamous-cell carcinoma (cT3-T4 or N1-N3 M0) were treated with neoadjuvant radiochemotherapy following the CROSS protocol [10]. Patients with locally advanced, non-metastatic esophageal adenocarcinoma (cT3-T4 or N1-N3 M0) received perioperative chemotherapy following the FLOT protocol or neoadjuvant chemoradiotherapy [14]. If the pathological examination of the resected tissue unexpectedly revealed a locally advanced esophageal adenocarcinoma (cT3-T4 or N1-N3 MX), as opposed to the preoperative staging, adjuvant chemotherapy was administered. Patients who refused neoadjuvant radiochemotherapy or perioperative chemotherapy primarily underwent surgery. The UKE is a high-volume center for the treatment of esophageal carcinomas certified by the German Cancer Society.

### 2.3. Definition of Socioeconomic Status

As in previous studies, the purchasing power for the postal codes in the patients’ addresses was used as a surrogate parameter for SES [15,16,17]. The purchasing power data were acquired from Michael Bauer Research GmbH. Purchasing power reflects net household income. It encompasses all income from labor, capital investment, rents, leases after taxes and social security contributions and also includes transfers such as unemployment benefits, child allowances, and pensions. Income has previously been certified to correctly indicate socioeconomic status [17,18,19,20]. Based on the German GEDA study, a cut-off of EUR 24,000 per year was set as the threshold for a low or high socioeconomic status [21].

### 2.4. Further Data Retrieval

As part of the preoperative work-up, known comorbidities and typical risk factors such as nicotine or alcohol abuse were routinely recorded in our Clinical Information System (Soarian Clinicals^®^, Cerner, Kansas City, MO, USA). Nicotine abuse was defined as the consumption of more than 10 packyears. The number of packyears has been calculated by multiplying the number of cigarette packs smoked per day by the number of years estimated by the patient. Alcohol abuse was present when the patient, currently or in the history, fulfilled the general criteria of the dependence syndrome according to the Diagnostic and Statistical Manual of Mental Disorders, Fifth Edition [22]. If the patients agreed to data collection, the patient and treatment characteristics were transferred from the Clinical Information System to our scientific database system (Ninox^®^, Berlin, Germany). Based on this, the preoperative Charlson Comorbidity Index before surgery was raised [23]. Postoperative complications were recorded as defined by the Esophagectomy Complications Consensus Group (ECCG) and classified according to Clavien–Dindo [24,25]. For overall survival, the time was recorded from the date of surgery to either the date of death of any cause according to the German National Population Register or the date of last access to the population register (last follow-up). The resulting median follow-up time was 20 months (range 0–108 months).

### 2.5. Statistical Analysis

The Statistical Package for Social Sciences (SPSS^®^) for Mac (Version 28) was used for the statistical analysis. Median and interquartile range (IQR) was used to describe the distribution of continuous and ordinal variables, and percentages were used for categorical variables. The chi-squared test was used to determine the univariate association between categorical variables. The Mann–Whitney U test was performed to compare continuous variables.

Survival curves for the overall survival of the patients were plotted (according to the Kaplan–Meier method) and analyzed by implementing the log-rank test as a univariate model. Cox proportional hazard regression was performed as a multivariate model of survival. The Cox proportional hazard regression model satisfied the proportional hazards assumptions. As variable entering method “Enter” was used. A *p*-value < 0.05 was considered to be statistically significant.

## 3. Results

### 3.1. Associations of Socioeconomic Status and Cohort Characteristics

The associations of SES with the cohort’s relevant clinical and pathological characteristics are shown in Table 1. The median estimated annual income of all 310 patients in the study was EUR 24,145 (IQR 21,399–26,474). A total of 154 patients (49.7%) with an estimated annual income of less than or equal to EUR 24,000 were assigned to the low SES group. Conversely, 156 patients (50.3%) with an estimated annual income of more than EUR 24,000 were assigned to the high SES group. The median annual income of low SES patients amounted to EUR 21,399 (IQR 20,057–22,496), and that of high SES patients was EUR 26,452 (24,717–28,293) per year. Regarding the type of health insurance, 260 patients (83.9%) had SHI, and 50 patients were privately insured (16.1%). As expected, patients with high SES were significantly more likely to have PHI (*p* = 0.035).

The median age was 64 (IQR 57–72) and showed no association with SES (*p* = 0.120). Likewise, the distribution of, in total, 242 men (78.1%) and 68 (21.9%) women did not differ between high and low SES (*p* = 0.445). Low SES patients were more likely to be smokers (*p* = 0.040) and tended towards a higher Body Mass Index (BMI, 25.2 (IQR 22.7–29.0) kg/m^2^, *p* = 0.054) compared to high SES patients (24.9 (IQR 21.8–27.5) kg/m^2^, *p* = 0.054). Regarding alcohol abuse, no significant difference was found between the two groups (*p* = 0.577). The mean Charlson Comorbidity Index (CCI) of all patients at the time of surgery was 4 (IQR 3–5). Interestingly, the CCI did not differ significantly between low and high SES (*p* = 0.815).

Concerning histological subtypes, patients with adenocarcinoma and squamous cell carcinoma were approximately equally distributed between the groups (*p* = 0.965). Regarding neoadjuvant treatment, 64 patients (20.6%) received chemotherapy according to the FLOT protocol, and 79 (25.5%) received radiochemotherapy following the CROSS protocol. The frequency of neoadjuvant therapy did not differ significantly between high and low SES (*p* = 0.662).

Open surgery was performed in 139 patients (44.8%) and 89 (28.7%) underwent laparoscopic surgery. Hybrid procedures (laparoscopic abdominal surgery, open thoracic surgery) were performed in 48 patients (15.5%), and 34 patients (11.0%) underwent complete robotic esophagectomy. No differences were found between high and low SES in the frequency of surgical techniques used (*p* = 0.839). Ninety patients (29.0%) developed a severe postoperative complication (Clavien–Dindo grades IIIb–IVb) and forty-four patients (14.2%) died during the hospital stay. The severity and frequency of postoperative complications (Clavien–Dindo) and in-hospital mortality did not differ between high and low SES (*p* = 0.443).

The UICC tumor stage also did not differ between groups (*p* = 0.542). Likewise, there was no significant difference in pathological tumor staging (*p* = 0.490), lymph node staging (*p* = 0.433), and resection status either (*p* = 0.284). Additionally, no differences were found in the frequency of adjuvant radiotherapy (*p* = 0.845) or chemotherapy between high and low SES (*p* = 0.582).

### 3.2. Associations of Socioeconomic Status and Survival

**Figure 1:**
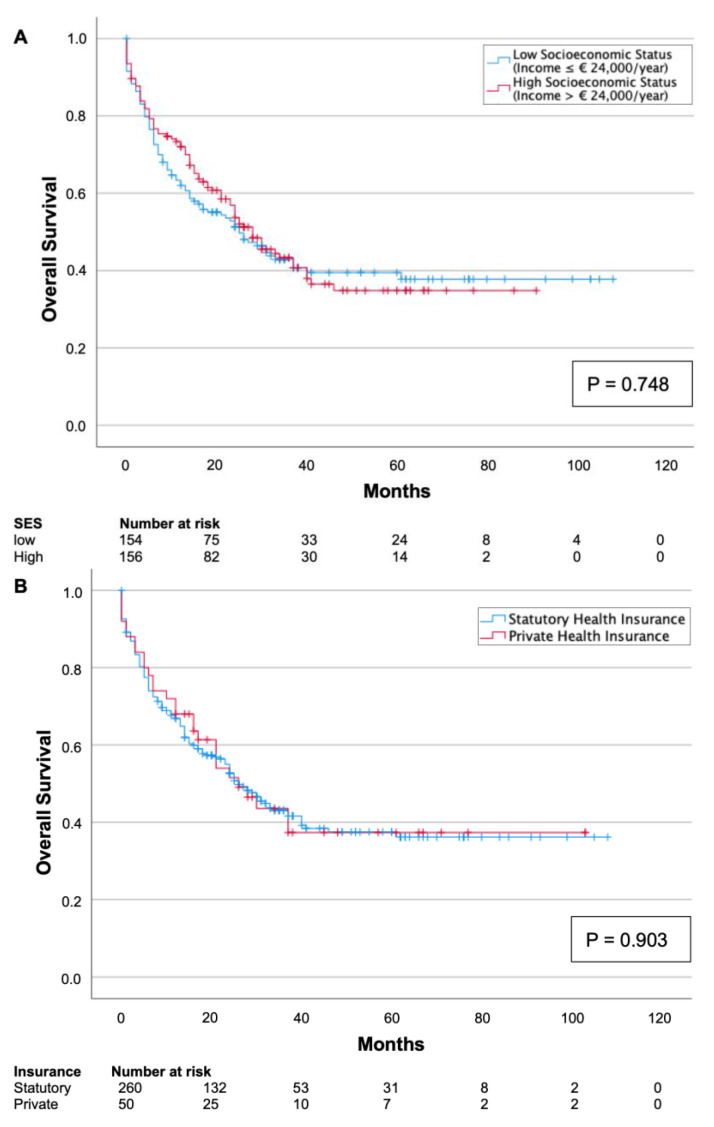
Kaplan–Meier survival curves split by low and high socioeconomic status (**A**) and type of health insurance (**B**). Neither socioeconomic status (*p* = 0.748) nor type of health insurance (*p* = 0.903) was associated with overall survival.

Univariate survival analysis revealed no association between socioeconomic status and survival (*p* = 0.748, Figure 1A). Similarly, the type of health insurance (SHI vs. PHI) was not associated with overall survival (*p* = 0.903, Figure 1B).

**Figure 2:**
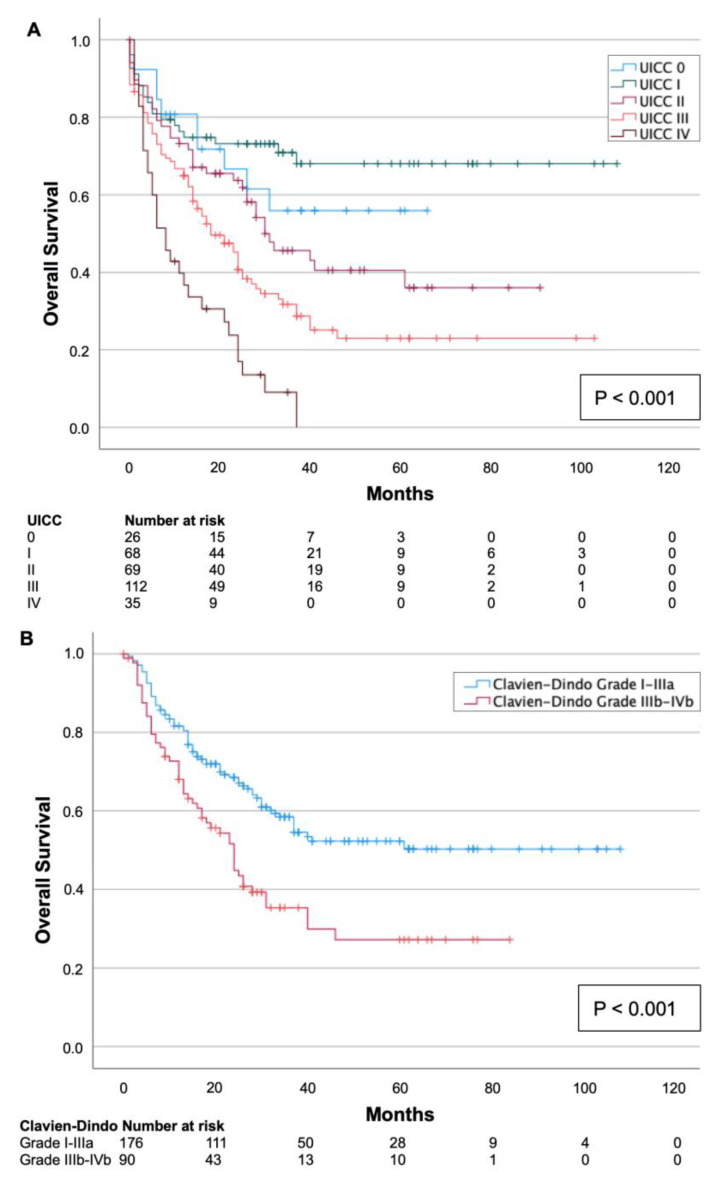
Kaplan–Meier survival curves split by UICC tumor stage (**A**) and severity of postoperative complication according to Clavien–Dindo (**B**). An advanced tumor stage and severe postoperative complication (Clavien–Dindo grade IIIb–IVb) were related to shorter overall survival (*p* < 0.001).

As expected, univariate survival analysis confirmed a significant association with the CCI (*p* = 0.007, Table 2): The median survival of patients with a low CCI (≤4) was 50 (95%CI 44–57) months, whereas the median survival for patients with a high CCI (>5) was 42 (95%CI 34–51) months. Moreover, patients who underwent laparoscopic surgery showed significantly longer survival in univariate analysis with 59 (95%CI 49–56) months compared to patients who underwent open surgery (*p* = 0.014, Table 2). Additionally, patients with an advanced tumor stage showed poorer survival: the median survival for patients with UICC stage IV was 13 (95%CI 9–17) months, while the median survival for patients with UICC stage I was 77 (95%CI 66–89) months (*p* < 0.001, Figure 2A). Furthermore, the median survival of patients with minor postoperative complications (Clavien–Dindo grades I–IIIa) was more favorable (65 months (95%CI 57–72)) than the survival of patients with severe postoperative complications (Clavien–Dindo grades IIB–IVb) with a median of 36 (95%CI 28–43) months (*p* < 0.001, Figure 2B). No significant difference in survival was observed for the histological type (adenocarcinoma vs. squamous-cell carcinoma, *p* = 0.325, Table 2).

## 4. Discussion

The present retrospective single-center study was the first study conducted in Germany to examine whether SES is associated with treatment outcomes after oncologic esophagectomy: First, it was found that socioeconomically deprived esophageal cancer patients were not more likely to have advanced tumor stages or severe comorbidities at the time of surgery. Second, no associations were found between SES and preoperative treatment, surgical technique, postoperative complications, in-hospital mortality, or adjuvant therapy. Last, neither SES nor the type of health insurance (SHI vs. PHI) had an influence on overall survival.

As no German study on this issue has been published so far, the results of the present study can only be compared with studies from other countries with considerably different healthcare systems. Those previous studies are usually based on national cancer registries and therefore have a higher number of patients. However, the analysis of registries is often accompanied by the problem that detailed information—for example, comorbidities, surgical technique, and postoperative complications—has not been systematically recorded and these details are, therefore, missing as confounders in the analysis.

For example, a French study using data from the French Network of Cancer Registries (FRANCIM) was published in 2021 and included 3250 esophageal cancer patients. It was found that the prognosis of esophageal cancer patients was markedly worsened by socioeconomic deprivation. More precisely, the hazard ratio of death in the lowest socioeconomic quintile compared to the highest quintile was 1.44 (95%CI 1.13–183). In this study, the tumor stage, tumor stage, comorbidities, surgical technique, and severity of postoperative complications were not considered as confounders in the analysis [26].

An analysis of Canadian databases, including 2125 esophageal adenocarcinoma patients diagnosed between 1993 and 2012, showed no association between SES and tumor stage at diagnosis or conducted therapy (surgery, chemotherapy, or radiotherapy). Moreover, the multivariate survival analysis showed no association between SES and the survival of patients with esophageal adenocarcinoma. In this study, comorbidity was estimated using the Johns Hopkins Adjusted Clinical Groups (ACG) case-mix system. Again, detailed information on the performed treatment, such as surgical technique and the severity of postoperative complications, was lacking in this study [27].

An analysis of the UK Hospital Episode Statistics (HES) database included 6282 patients who had received esophagectomy for cancer from 1998–2002. The authors found that the highest levels of socioeconomic deprivation had significantly higher mortality rates than those in areas with lower levels of deprivation. Precisely, the 30-day risk of death in patients in the lowest quintile for deprivation after esophagectomy was increased 1.37-fold (95%CI 1.03–1.85) compared to the highest quintile. However, in this study, important confounders are unknown to the authors, e.g., no records were available on whether the surgery was performed with palliative or curative intent. [28].

This study analyzed 4097 patients from Taiwan’s National Health Insurance Research Database (NHIRD) diagnosed with esophageal cancer who underwent any hospital treatment for their disease between 2002 and 2006. Here, the authors distinguished between individual SES based on the enrollee category and neighborhood SES. It was found that 5-year overall survival rates were poorest amongst individuals with low individual SES living in deprived neighborhoods. Although Taiwan has a universal healthcare system, patients with high individual SES from deprived areas were more likely to undergo surgery [29].

In a study from the USA, published in 2017, 11,599 patients from the National Cancer Data Base (NCDB) who underwent esophagectomy for cancer between 2003 and 2011 were analyzed. The multivariate analysis, which included tumor stage and the Charlson comorbidity index, showed that patients in the highest income quartile had better overall survival than those in the lowest quartile (HR 0.803, 95%CI 0.743–0.867) [30].

In a further study from the USA, which evaluated 60,621 patients with UICC stages I–III between 2004 and 2015, results demonstrated that black patients, uninsured patients, and patients living in areas with lower levels of education receive surgical interventions or any other kind of therapy less often. Consequently, patients receiving surgical treatment, compared to both, no treatment and definitive chemoradiation, had significantly better long-term survival. However, the researchers did not investigate whether socioeconomically disadvantaged patients within the group of surgically treated patients had a worse outcome [31].

A Dutch study by Bus et al. based on the Eindhoven Cancer Registry (ECR) found that patients with low SES were diagnosed with a more advanced tumor stage (13% vs. 10%, stage T4). The researchers found no significant difference in survival within the curative treatment group, which included 708 patients. The authors systematically recorded the comorbidity using the Charlson Comorbidity Index; relevant factors such as surgical technique and severity of postoperative complications were not recorded [32].

A limitation of the present study is its nature as a single-center study: First, SES had no significant influence on the outcome of patients treated at only one single high-volume center. Second, the number of patients is low compared to the above-mentioned register of studies from other countries. Therefore, a minor association between SES and postoperative outcomes cannot be ruled out with certainty. Of course, an evaluation at the German national level would be desirable to increase the number of cases and include all relevant institutions. However, unfortunately, as important confounders were not systematically recorded in the German national cancer registry until today, the authors do not believe this is feasible in the near future.

## 5. Conclusions

Once socioeconomically deprived patients gained access to the treatment at a high-volume center, fortunately, no significant differences in treatment outcomes compared to socioeconomically privileged patients were found.

## Figures and Tables

**Table 1:** Associations of socioeconomic status and cohort characteristics. Data are given as median (interquartile range (IQR)) or N (%).

Characteristics	All Patients (N = 310)	Low Socioeconomic Status (Income ≤ EUR 24,000/Year, N = 154)	High Socioeconomic Status (Income > EUR 24,000/year, N = 156)	*p*-Value
**Age** [years (IQR)]	64 (57–72)	63 (56–70)	67 (58–72)	0.120
**Sex** [n (%)]				0.445
Male	242 (78.1)	123 (39.7)	119 (38.4)	
Female	68 (21.9)	31 (10.0)	37 (11.9)	
**Average income** [EUR/year (IQR)]	24,145 (21,399–26,474)	21,399 (20,057–22,496)	26,452 (24,717–28,293)	**<0.001**
**Type of health insurance**				
Statutory health insurance (SHI)	260 (83.9)	136 (43.9)	124 (40.0)	**0.035**
Private health insurance (PHI)	50 (16.1)	18 (5.8)	32 (10.3)	
**Charlson Comorbidity Index** (IQR)	4 (3–5)	4 (3–5)	4 (3–6)	0.815
**Nicotine abuse**				**0.040**
Yes	133 (42.9)	75 (24.2)	58 (18.7)	
No	177 (57.1)	79 (25.5)	98 (31.6)	
**Alcohol abuse**				0.577
Yes	52 (16.8)	24 (7.7)	28 (9.0)	
No	258 (83.2)	130 (41.9)	128 (41.3)	
**BMI** (kg/m^2^(IQR)	25.1 (22.2–28.1)	25.2 (22.7–29.0)	24.9 (21.8–27.5)	0.054
**Entity** [n (%)]				0.965
Adenocarcinoma	204 (65.8)	100 (32.3)	104 (33.5)	
Squamous cell carcinoma	99 (31.9)	50 (16.1)	49 (15.8)	
Other	7 (2.3)	4 (1.3)	3 (1.0)	
**Preoperative treatment** [n (%)]				0.662
FLOT	64 (20.6)	36 (11.6)	28 (9.0)	
CROSS	79 (25.5)	28 (9.0)	41 (13.2)	
Definitive radiochemotherapy	15 (4.8)	7 (2.3)	7 (2.3)	
None	152 (49.0)	72 (23.2)	80 (25.2)	
**Type of access** [n (%)]				0.839
Open	139 (44.8)	70 (22.6)	69 (22.3)	
Laparoscopic	89 (28.7)	46 (14.8)	43 (13.9)	
Hybrid	48 (15.5)	21 (6.8)	27 (8.7)	
Robotic	34 (11.0)	17 (5.5)	17 (5.5)	
**UICC classification 8th edition** [n (%)]				0.542
0	26 (8.4)	12 (3.9)	14 (4.5)	
I	68 (21.9)	38 (12.3)	30 (9.7)	
II	69 (22.3)	34 (11.0)	35 (11.3)	
III	112 (36.1)	50 (16.1)	62 (20)	
IV	35 (11.3)	20 (6.5)	15 (4.8)	
**Tumor staging (pT)** [n (%)]				0.490
pT0	36 (11.6)	18 (5.8)	18 (5.8)	
pT1a	20 (6.5)	12 (3.9)	8 (2.6)	
pT1b	59 (19.0)	30 (9.7)	29 (9.4)	
pT2	50 (16.1)	27 (8.7)	23 (7.4)	
pT3	137 (44.2)	61 (19.7)	76 (24.5)	
pT4a	6 (1.9)	4 (1.3)	2 (0.6)	
pT4b	2 (0.6)	2 (0.6)	0 (0)	
**Lymph node staging (pN)** [n (%)]				0.433
pN0	157 (50.6)	84 (27.1)	73 (23.5)	
pN1	65 (21.0)	32 (10.3)	33 (10.6)	
pN2	54 (17.4)	22 (7.1)	32 (10.3)	
pN3	34 (11.0)	16 (5.2)	18 (5.8)	
**Resection status** [n (%)]				0.284
R0	285 (91.9)	144 (46.5)	141 (45.5)	
R1	8 (7.7)	9 (2.9)	15 (4.8)	
R2	1 (0.3)	1 (0.3)	0 (0)	
**Clavien–Dindo Classification** [n (%)]				0.443
I	73 (23.5)	40 (12.9)	33 (10.6)	
II	73 (23.5)	36 (11.6)	37 (11.9)	
IIIa	30 (9.7)	9 (2.9)	21 (6.8)	
IIIb	37 (11.9)	20 (6.5)	12 (3.9)	
IVa	45 (14.5)	22 (7.1)	23 (7.4)	
IVb	8 (2.6)	4 (1.3)	4 (1.3)	
V (in-hospital death)	44 (14.2)	23 (7.4)	21 (6.8)	
**Adjuvant treatment** [n (%)]				
Radiotherapy	21 (6.8)	10 (3.2)	11 (3.5)	0.845
Chemotherapy (including FLOT)	91 (29.4)	43 (13.9)	48 (15.5)	0.582

**Table 2:** Univariate and multivariate survival analysis.

Characteristics	N (%)	Univariate Analysis		Multivariate Analysis	
		Median Survival in Months (95% CI)	*p*-Value (Log-Rank Test)	Hazard Ratio (95% CI)	*p*-Value (Cox Regression)
**Age**			**0.015**		0.666
<50	32 (10.3)	53 (36–71)		-	
50–59	75 (24.2)	55 (44–66)		1.21 (0.60–2,44)	
60–69	106 (34.2)	49 (39–58)		1.25 (0.64–2.44)	
70–79	78 (25.2)	44 (33–55)		1.87 (0.72–4.87)	
≥80	19 (6.1)	19 (8–30)		2.21 (0.74–6.61)	
**Gender** [n (%)]			0.590		-
Male	242 (78.1)	50 (44–57)		-	
Female	68 (21.9)	38 (29–47)		-	
**Socioeconomic Status**			0.748		0.454
Low SES (Income ≤ EUR 24,000/year)		50 (42–58)		-	
High SES (Income > EUR 24,000/year)		43 (36–49)		0.86 (0.59–1.27)	
**Type of health insurance**			0.903		-
Statutory health insurance (SHI)	260 (83.9)	49 (43–55)		-	
Private health insurance (PHI)	50 (16.1)	48 (35–61)		-	
**Charlson Comorbidity Index** (SD)			**0.007**		0.879
Low (≤4)		54 (47–62)		-	
High (>5)		42 (34–51)		1.05 (0.53–2,08)	
**Entity** [n (%)]			0.325		-
Adenocarcinoma	204 (65.8)	50 (43–57)		-	
Squamous cell carcinoma	99 (31.9)	45 (36–54)		-	
**Type of access** [n (%)]			**0.014**		0.06
Open	139 (44.8)	41 (33–48)		-	
Laparoscopic	89 (28.7)	59 (49–69)		0.54 (0.34–0.68)	
Hybrid	48 (15.5)	53 (39–68)		0.72 (0.41–1.24)	
Robotic	34 (11.0)	35 (26–44)		0.72 (0.40–1.31)	
**UICC classification 8th edition** [n (%)]			**<0.001**		**<0.001**
0	26 (8.4)	44 (33–55)		-	
I	68 (21.9)	77 (66–89)		0.39 (0.16–0.95)	
II	69 (22.3)	46 (36–55)		1.10 (0.51–2.38)	
III	112 (36.1)	36 (28–44)		1.73 (0.84–3.57)	
IV	35 (11.3)	13 (9–17)		4.16 (1.87–9.28)	
**Resection status** [n (%)]			**<0.001**		0.052
R0	285 (91.9)	51 (45–57)		-	
R1	8 (7.7)	20 (10–30)		1.83 (0.99–3.37)	
R2	1 (0.3)	1 (1–1)		-	
**Clavien–Dindo Classification** [n (%)]			**<0.001**		**0.001**
I–IIIa	175 (56.5)	65 (57–72)		-	
IIIb–IVb	90 (29)	36 (28–43)		1.84 (1.27–2.67)	

## Data Availability

The data presented in this study are available on request from the corresponding author.
